# Evaluating the biocontrol potential of Canadian strain *Bacillus velezensis* 1B-23 via its surfactin production at various pHs and temperatures

**DOI:** 10.1186/s12896-021-00690-x

**Published:** 2021-04-29

**Authors:** Michelle S. M. Li, David A. Piccoli, Tim McDowell, Jacqueline MacDonald, Justin Renaud, Ze-Chun Yuan

**Affiliations:** 1 Department of Microbiology and Immunology, University of Western Ontario, 1151 Richmond Street, London, Ontario N6A 5B7 Canada; 2 London Research and Development Centre, Agriculture and Agri-Food Canada, 1391 Sandford Street, London, Ontario N5V 4T3 Canada

**Keywords:** Antibiotic, Antifungal, Antimicrobial, *Bacillus*, Biocontrol, Biological control, Biopesticide, Biosurfactant, Microbial pesticide, Phytopathogen, Surfactin

## Abstract

**Background:**

Microorganisms, including *Bacillus* species are used to help control plant pathogens, thereby reducing reliance on synthetic pesticides in agriculture. *Bacillus velezensis* strain 1B-23 has been shown to reduce symptoms of bacterial disease caused by *Clavibacter michiganensis* subsp. *michiganensis* in greenhouse-grown tomatoes, with in vitro studies implicating the lipopeptide surfactin as a key antimicrobial. While surfactin is known to be effective against many bacterial pathogens, it is inhibitory to a smaller proportion of fungi which nonetheless cause the majority of crop diseases. In addition, knowledge of optimal conditions for surfactin production in *B. velezensis* is lacking.

**Results:**

Here, *B. velezensis* 1B-23 was shown to inhibit in vitro growth of 10 fungal strains including *Candida albicans*, *Cochliobolus carbonum*, *Cryptococcus neoformans*, *Cylindrocarpon destructans Fusarium oxysporum*, *Fusarium solani*, *Monilinia fructicola*, and *Rhizoctonia solani*, as well as two strains of *C. michiganensis michiganensis*. Three of the fungal strains (*C. carbonum*, *C. neoformans*, and *M. fructicola*) and the bacterial strains were also inhibited by purified surfactin (surfactin C, or [Leu7] surfactin C15) from *B. velezensis* 1B-23. Optimal surfactin production occurred in vitro at a relatively low temperature (16 °C) and a slightly acidic pH of 6.0. In addition to surfactin, *B. velenzensis* also produced macrolactins, cyclic dipeptides and minor amounts of iturins which could be responsible for the bioactivity against fungal strains which were not inhibited by purified surfactin C.

**Conclusions:**

Our study indicates that *B. velezensis* 1B-23 has potential as a biocontrol agent against both bacterial and fungal pathogens, and may be particularly useful in slightly acidic soils of cooler climates.

**Supplementary Information:**

The online version contains supplementary material available at 10.1186/s12896-021-00690-x.

## Background

An increasing burden is being placed on agricultural industries to sustain earth’s rapidly growing population [1, 2]. By 2050, the world’s population is expected to exceed 9 billion, requiring by some estimates, a 70 to 100% increase in global food production [1]. As a result, intensified and sustainable agricultural strategies will be required to keep up with the growing population; however, the amount of agriculturally available and usable land is becoming restricted and further complicated by ongoing climate change [1]. One opportunity for increasing or maintaining production from existing land is to target plant pathogens that lead to disease in crops or in the humans or animals that consume them. Protecting crops against pathogens can help increase productivity by reducing direct losses and product recalls.

By preventing and controlling agricultural disease, farmers can limit crop waste while maximizing their yields [3]. While synthetic pesticides are effective to control pests and pathogens, these chemical agents are associated with multiple environmental challenges including development of resistance in the pathogens, bioaccumulation of toxic substances, pollution and overall worsening of soil fertility [4–6]. As a result, researchers and farmers are seeking more ecofriendly methods to prevent these issues.

One such method is the use of microbial pesticides, a type of biological control (or biocontrol) which uses microorganisms to reduce agricultural disease. These microorganisms contribute to the control of phytopathogens through competitive inhibition, antimicrobial production, biofilm formation, interfering with cell-cell communication, and/or induction of systemic resistance, often in addition to promoting plant growth through biofertilization and root growth stimulation [7, 8]. In terms of environmental effects, microbial pesticides are preferable to synthetic pesticides because they are fully biodegradable and often produce numerous antimicrobial compounds, reducing the risk of developing pesticide resistance in the pathogen population [5].

Unfortunately, as compared to their synthetic counterparts, microbial pesticides exhibit variable effectiveness across different environments [9]. Including a diversity of microbial strains in one product increases the chance that at least some of them will remain active under any given condition. In fact, effective microbial pesticides often incorporate several complimentary bacterial strains (a ‘consortium’) that are suitable to the target plants, the region, and the pathogen(s) to be controlled. However, the strains within a consortium must be carefully selected to avoid competition among them [10] and the issue of inconsistent field performance remains, particularly in colder climates [8]. This further highlights the need to develop multiple strains that are adapted to different environments. New strains can be studied to determine the key factors that contribute to successful biocontrol under in vitro conditions to prescreen for suitability to the targeted plants, climate region, and ecosystem prior to plant or field studies [11].

Among the most investigated biocontrol agents, Gram-positive, rod-shaped *Bacillus* species are often included as one of the microorganisms in commercially-developed biopesticides [10, 12] These plant growth-promoting rhizobacteria are well-known for their biocontrol potential due to their extensive root colonization and biofilm formation, induction of systemic resistance and production of a wide range of secondary metabolites that contribute to their antimicrobial activity [13–15]. For instance, surfactin, iturin, fengycin, mycosubtilin and bacillomycin are secondary metabolite, biosurfactant lipopeptides that are often produced by *Bacillus* strains and are well known for their biocontrol properties [10, 16].

Surfactin is an amphiphilic and cyclic lipoheptapeptide that structurally resembles many traditional surfactants; it is a surface-active molecule that can decrease surface and interfacial tension. It can also increase the surface area of hydrophobic compounds, thereby increasing their bioavailability. Its natural functions include involvement in the formation of spore-developing structures and biofilm, swarming motility, quorum sensing, and antimicrobial activity [17]. It is a secondary metabolite that is synthesized non-ribosomally, and as such, is not essential for growth of the organism [18, 19].

Surfactin is advantageous as a biopesticide component due to its stability over a wide range of temperatures and pHs, in addition to the biodegradability and low toxicity that is common to many natural antimicrobials [20]. As an antimicrobial agent, surfactin inserts itself into cell bilayers, chelates cations and solubilizes membranes, and lyses pathogens through pore formation [21]. Yet even without pathogen lysis, surfactin can contribute to biocontrol via its involvement in *Bacillus* biofilm formation. This biofilm formation can disrupt the biofilm of cohabitant pathogens, and can also induce systemic resistance in plants [13, 15].

The surfactin-producing *Bacillus* species include *Bacillus subtilis*, *Bacillus amyloliquefaciens*, and *Bacillus velezensis* [10]. *B. subtilis* is the most frequently studied species with respect to surfactin production, and recent studies have aimed to determine the optimal temperature and/or pH for surfactin production in various strains. For example, the highest levels of surfactin production was found to be between 30 °C and 37 °C and at pH between 7 and 9 for *B. subtilis* strain KLP2015 [22], 36 °C for *B. subtilis* strain UFPEDA 438 [23], and 37 °C for *B. subtilis* strain NLIM 0110 [24]. In contrast, *B. amyloliquefaciens* strain 629 exhibited its highest surfactin production at the much lower temperature of 15 °C [25]. *B. velezensis* is most closely related to *B. amyloliquefaciens* [26], suggesting that its surfactin production may be highest at lower temperatures. To our knowledge, neither temperature- nor pH-specific surfactin production has been studied in *B. velezensis*. While Madhaiyan et al. [27] reported that optimal growth of *B. velezensis* strain CBMB205^T^ (formerly *Bacillus methylotrophicus*) occurred at 28 °C and pH 7, conditions that are optimal for growth are not necessarily optimal for surfactin production.

Our previous research shows that *B. velezensis* strain 1B-23, a crude extract of its hydrophobic metabolites, and its isolated surfactin have antimicrobial properties in vitro against the Gram-positive plant pathogen *Clavibacter michiganensis* subsp. *michiganensis*. In addition, greenhouse-grown tomatoes infected with this pathogen showed reduced symptoms when treated with *B. velezensis* 1B-23 [28]. While surfactin is known to be effective against many bacteria, it is inhibitory to a smaller proportion of fungi [21] which nonetheless cause the majority of crop diseases. The current study therefore aimed to confirm the results of antimicrobial activity against *C. michiganensis michiganensis*, to explore the antimicrobial activity of *B. velezensis* 1B-23 and its surfactin against fungal pathogens, and to determine the optimal temperature and pH for production of surfactin.

## Results

### Antimicrobial activity of 1B-23, its crude extract & surfactin

In a previous in vitro study, *B. velezensis* 1B-23 and its surfactin were shown to effectively inhibit growth of *Clavibacter michiganensis* subsp. *michiganensis* strain 98–1, but not *Pseudomonas syringae* DC3000 [28]. This *B. velezensis* strain also demonstrated its ability to suppress the disease symptoms caused by *C. michiganensis michiganensis* 98–1 in tomato plants [28]. *C. michiganensis michiganensis* is a Gram positive bacterium considered to be one of the most destructive diseases of tomato [29], while *P. syringae* DC3000 is a Gram negative pathogen that affects tomato, *Arabidopsis*, and *Nicotiana* [30].

To test the in vitro activity of *B. velezensis* 1B-23 against additional pathogens, filter discs inoculated with 1B-23 were placed onto LB agar plates cultured with pathogenic bacteria or fungi (Table 1). *B. velezensis* 1B-23 inhibited growth of all 12 tested strains representing 9 species, as evidenced by zones of inhibition, or clearance, surrounding the filter discs (Additional file 1).

**Table 1:** In vitro antimicrobial capability of 1B-23 culture and surfactin against selected pathogens

Pathogen	1B-23	Crude extract	MIC / mg/mL	Surfactin	MIC / mg/mL
*Clavibacter michiganensis* 98–1	+	+	5	+	1
*Clavibacter michiganensis* JD83–1	+	+	1	+	1
*Rhizoctonia solani*	+	–	None^a^	–	None^a^
*Monilinia fructicola*	Inconclusive	+	1	+	1
*Cylindrocarpon destructans* 1666	+	–	None^a^	–	None^a^
*Cochliobolus carbonum*	+	+	5	+	1
*Fusarium solani*	+	–	None^a^	–	None^a^
*Fusarium oxysporum*	+	–	None^a^	–	None^a^
*Cryptococcus neoformans var. grubii* H99	+	+	10	–	None^a^
*Cryptococcus neoformans var. neoformans* JEC20	+	+	10	–	None^a^
*Cryptococcus neoformans var. neoformans* Y290.90	+	+	10	+	1
*Candida albicans* ARG100	+	–	None^a^	–	None^a^

Degree of inhibition zone of clearance: (+) indicates a visible zone of clearance created, (−) indicates no visible zone of clearance. *MIC* minimum inhibition concentration (the lowest tested concentration that inhibited visible microbial growth to any degree); ^a^ no effective MIC found for the tested concentration range (0.5 to 10 mg/mL)

Pathogen inhibition was then tested in a similar way using filter discs inoculated with a crude extract of hydrophobic metabolites that were isolated from *B. velezensis* 1B-23, rather than with the organism itself. This crude extract was expected to contain hydrophobic lipopeptide antimicrobials, which were putatively identified as surfactin B, surfactin C, and surfactin D (also called [Leu7] surfactin C14, [Leu7] surfactin C15, and [Leu7] surfactin C16, respectively), with smaller amounts of epoxy-macrolactin A, cyclic dipeptides, and minor amounts of iturin (Additional file 2). Inhibition by the extract would imply an effect of specific antimicrobial chemicals rather than more general mechanisms of inhibition, such as competition for resources. Of the 12 strains across 9 species that were inhibited by living 1B-23, 7 strains representing 4 species were also inhibited by at least one of the tested concentrations of crude extract (1.0 mg/mL, 2.5 mg/mL, 5.0 mg/mL, or 10 mg/mL; Table 1 and Additional file 1). Those not inhibited by the crude extract were: *R. solani, C. destructans* 1666, *F. solani*, *F. oxysporum*, and *C. albicans* ARG100*.*

We then used filter discs inoculated with isolated surfactin C from *B. velezensis* 1B-23 to test antimicrobial effects directly, since this surfactin (along with surfactins A and B) was previously implicated in the inhibition of the phytopathogen *C. michiganensis michiganensis* 98–1 [28]. Of the 7 strains (4 species) that were inhibited by the crude extract of hydrophobic metabolites, 5 strains representing 4 species were also inhibited by the isolated surfactin at a concentration of at least 1.0 mg/mL (Table 1 and Additional file 1). These included *C. michiganensis*, *Monilinia fructicola*, *Cochliobolus carbonum*, and one strain of *Cryptococcus neoformans*. Surfactin showed larger inhibition of *Clavibacter michiganensis* 98–1 and *C. neoformans var. neoformans* Y290.90, thereby indicating stronger biocontrol activity for these pathogens (Additional file 1).

In some cases, higher concentrations of crude extract were required to induce the same zone of clearance as the isolated surfactin (Table 1), indicating that the surfactin content is disproportionately responsible for the antimicrobial activity. Two strains of *C. neoformans*, as well as *Pseudomonas syringae* DC3000, were not inhibited by the isolated surfactin despite being inhibited by the crude extract.

### Temperature-specific Surfactin production

**Fig. 1:**
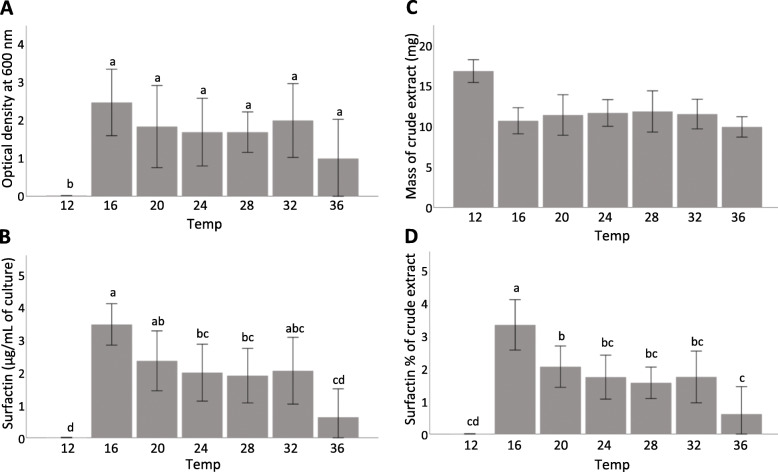
The effects of temperature at pH 6.8 on (**a**) 1B-23 growth, and (**b**) total surfactin production by 1B-23 where the mass of the product was semi-quantified using LC-MS. The average mass of crude extract obtained for each 100 mL sample (**c**) and the relative contribution of surfactin to the crude extract mass (**d**) are also shown. Results for each treatment are represented as the mean value of each replicate minus the abiotic negative control ± SD, *n* = 6. Means with the same letters are considered not statistically different (*P* < 0.05) according to Bonferoni’s post-hoc test

To test 1B-23’s total surfactin production at varying temperatures, we first measured the relative growth of 1B-23 cells and surfactin production after 72 h of growth at 12 °C, 16 °C, 20 °C, 24 °C, 28 °C, 32 °C, and 36 °C (Fig. 1a) at pH 6.8. The relative 1B-23 cell concentrations at 12 °C were very low, as indicated by optical density readings at 600 nm (OD_600_ = 0.085 ± 0.033; mean ± standard deviation), and were significanty different (*P* < 0.05) from each of the other temperature treatments. Between 16 °C and 32 °C, the average readings ranged from 1.67 ± 0.88 SD to 2.44 ± 0.87 SD, while at 36 °C the average dropped to 0.98 ± 1.0. However, none of the OD_600_ readings between 16 °C and 36 °C were statistically significantly different from each other.

Liquid chromatography coupled to high resolution mass spectrometry (LC-MS) was used to semi-quantify the amount of surfactin in these cell cultures. The surfactin present was measured from a small aliquot of crude extract, and values were extrapolated to estimate total amounts in initial cultures (Fig. 1b). As expected, surfactin production was the lowest at 12 °C (0.005 ± 0.005 μg/mL SD), where the measurable surfactin was detected in only one of the six replicates. This amount of surfactin at 12 °C was significanty lower than that at each of the other temperatures (*P* < 0.05). Significantly more surfactin was produced at 16 °C than at 12, 24, 28, and 36 °C.

We also calculated surfactin’s relative contribution to the crude extract mass to determine the contribution of surfactin to the total hydrophobic metabolites at these temperatures (Fig. 1c-d). At 12 °C, this relative contribution of surfactin was close to zero, and was not significantly different from the relative contributions between 24 °C and 36 °C. The relative surfactin contribution at 16 °C was highest, at 3.32 ± 0.31% SD, which was significanty greater than at 20 °C (2.05 ± 0.26%).

### Temperature-specific Surfactin production over time

**Fig. 2:**
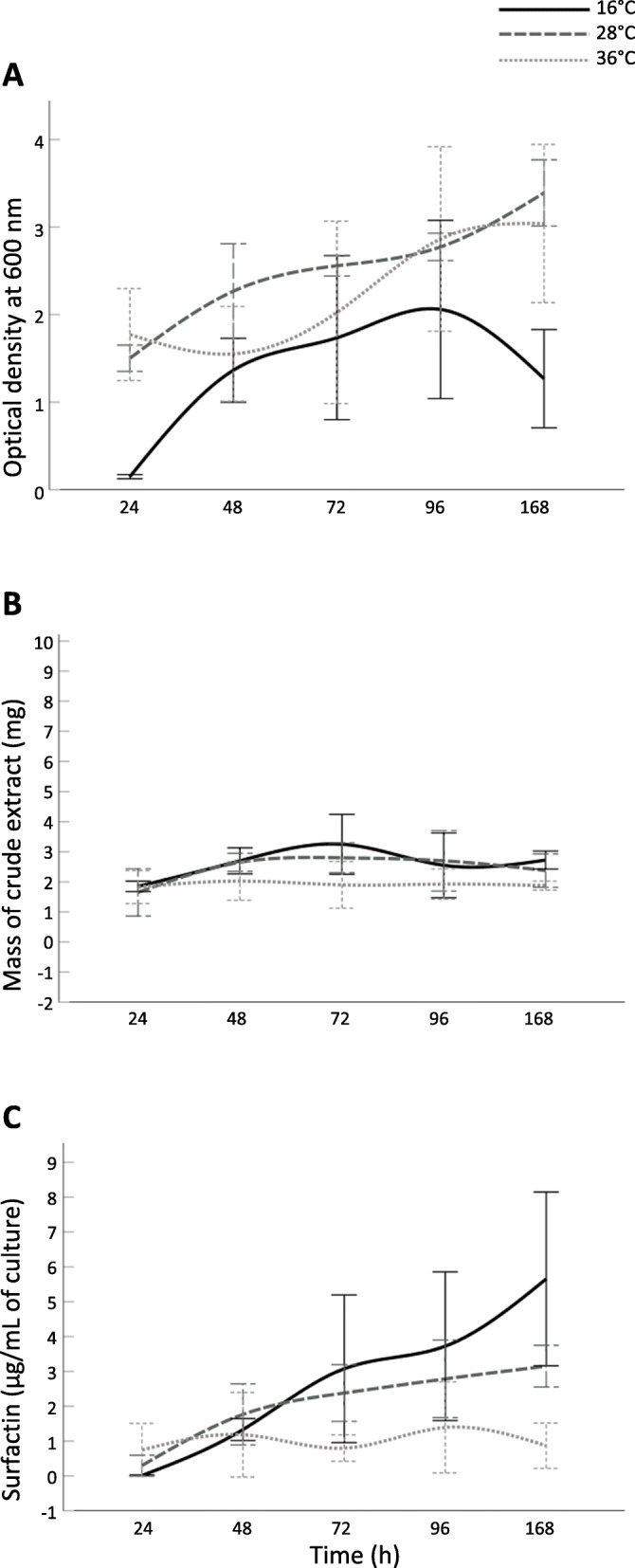
Effect of temperature on (**a**) 1B-23 culture growth, (**b**) crude extract mass per 10 mL culture and (**c**) total surfactin accumulation over time. *B. velezensis 1B-23* was grown at 16 °C (black, solid line), 28 °C (dark grey, dashed line) or 36 °C (light grey, dotted line) at pH 6.8 for a total of 7 days. The results for each treatment are represented as the mean value of each replicate minus the abiotic negative control ± SD, *n* = 4

To explore 1B-23’s growth and total surfactin production at varying temperatures over time, 3 representative temperatures from the previous assay were chosen for testing over a period of 7 days. Cell culture aliquots were extracted after 24, 48, 72, 96, and 168 h of incubation (Fig. 2).

Analysis of Variance (ANOVA) showed that both time and temperature were significant factors affecting the growth of the cultures (*p* = 0.000 and *p* = 0.011, respectively). The growth curves of 1B-23 at 28 °C and 36 °C were similar to one another, with the average OD_600_ values exceeding 1.5 after 24 h and exceeding 3.0 after 168 h. The OD_600_ values at 16 °C were similar to those at 28 °C and 36 °C between 48 and 96 h, but were significantly different from 28 °C and 36 °C at 24 h (*p* = 0.001 and *p* = 0.000, respectively) and 168 h (*p* = 0.004 and *p* = 0.012, respectively) (Fig. 2a).

The mass of crude hydrophobic metabolite extract (Fig. 2b) was similar across all time points (*p* > 0.05). However, overall values at 16 °C were detectably higher than those at 36 °C (*p* = 0.016), specifically after 168 h (*p* = 0.032). No statistically significant differences in crude extract amounts were detected between 16 °C and 28 °C, nor between 28 °C and 36 °C.

In contrast, time was a significant factor affecting surfactin accumulation in 1B-23 (Fig. 2c; *p* = 0.000). Overall, the amount of surfactin increased significantly between 24 and 48 h (*p* = 0.002), and between 48 and 96 h (*p* = 0.044), but not between 96 and 168 h. Temperature was not found to be a significant factor affecting surfactin accumulation in the time course data.

### pH-specific Surfactin production over time

**Fig. 3:**
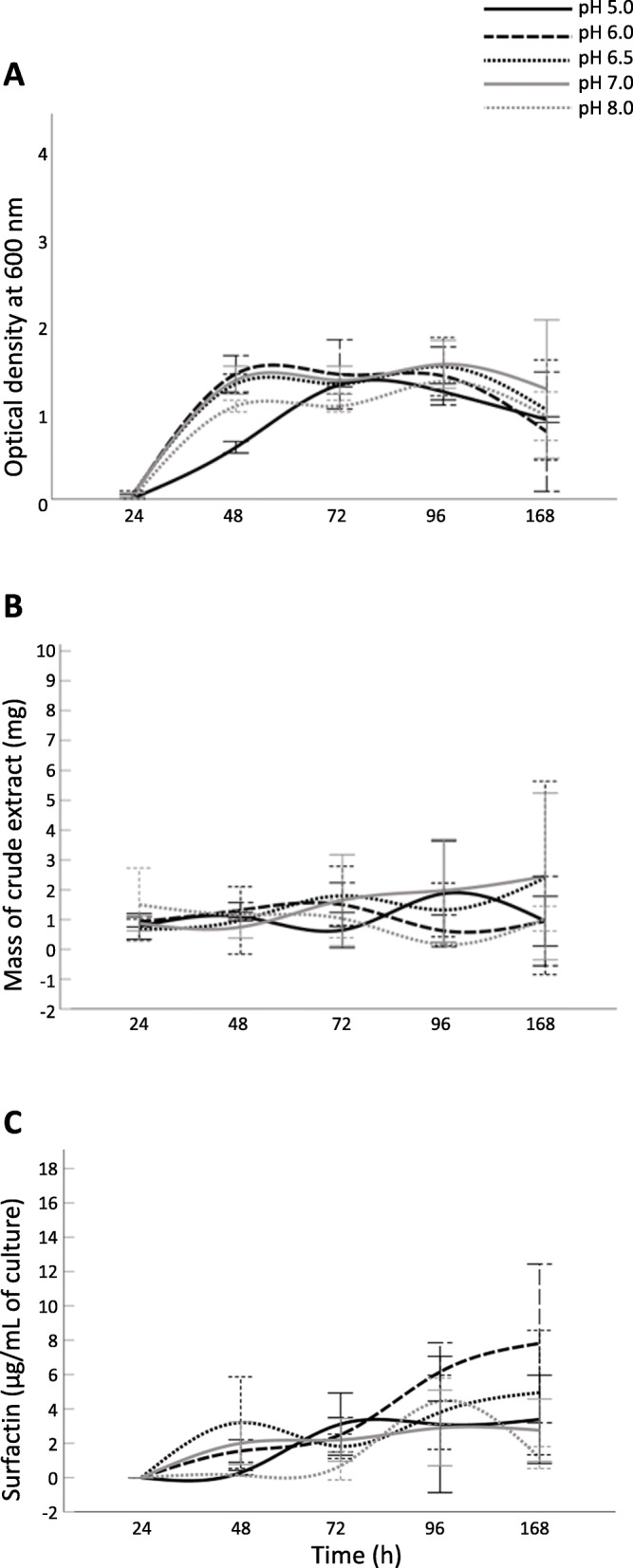
Effect of pH on (**a**) 1B-23 culture growth, (**b**) crude extract mass per 10 mL culture and (**c**) total surfactin accumulation over time. *B. velezensis 1B-23* was grown at pH 5.0 (black, solid line), pH 6.0 (black, dashed line), pH 6.5 (black, dotted line), pH 7.0 (grey, solid line), or pH 8.0 (grey, dotted line), at 16 °C. The results for each treatment are represented as the mean value of each replicate minus the abiotic negative control ± SD, *n* = 4

To explore 1B-23’s surfactin production while varying pH (over time), 5 pHs near the neutral range at the optimal surfactin production temperature (16 °C) were chosen for further examination. Similar to the temperature study, cell culture aliquots were extracted after 24, 48, 72, 96, and 168 h of incubation (Fig. 3).

The growth of the culture was significantly affected by time (*p* = 0.000) but not by pH (*p* > 0.05). The mass of crude hydrophobic metabolite extract was similar across all pHs and time points (Fig. 3a), with neither pH nor time detected as a statistically significant factor (*p >* 0.05).

Time was determined to be a significant factor that affected the amount of surfactin accumulation in our pH study (Fig. 3c; *p* = 0.000). Overall, the amount of surfactin increased significantly between 24 and 48 h (*p* = 0.006), and between 48 and 96 h (*p* = 0.003), but not between 96 and 168 h. This was the same pattern observed for the temperature time course (Fig. 2c). The only significant difference detected across pH conditions was between pH 6 and pH 8 overall (*p* = 0.030).

## Discussion

In the current study, *B. velezensis* 1B-23 cultures were effective at inhibiting the growth in a wide range of agriculturally relevant phytopathogens and other microbes. In fact, all of the tested organisms were inhibited by 1B-23 (Table 1). A subset of these organisms was also inhibited by the crude hydrophobic extract of 1B-23, which contains antimicrobials including macrolactins and the lipopeptide classes of surfactin and iturin. Within this subset of organisms, a further subset was inhibited by purified surfactin C alone.

Purified surfactin C at a concentration of 1 mg/mL inhibited growth of the two tested strains of *C. michiganensis* (Gram-positive bacterium), as well as three of the fungal test organisms: *M. fructicola* and *C. carbonum* (two unrelated ascomycete molds) and one strain of *C. neoformans* (a basidiomycete yeast). These organisms were also inhibited by the crude hydrophobic extract of 1B-23 at concentrations ranging from 1 to 10 mg/mL. In situations where surfactin and the crude extract were both able to inhibit growth at the same concentration (1 mg/mL), this indicated that other components of the crude extract are equally capable of growth inhibition as surfactin. Growth inhibition requiring a greater concentration of crude extract versus surfactin suggests that surfactin has stronger action against the microbe versus the averaged mixture of contents present within the crude extract. However, it cannot be concluded that surfactin is stronger than any specific antimicrobial from the crude extract (i.e. iturin, macrolactin, or cyclodipeptide), as we do not know the relative amounts of these in the extract.

In our study, it was only the two remaining strains of *C. neoformans* (a basidiomycete yeast) that were inhibited by the crude extract but not by surfactin. This indicates that the other crude extract components may perform better than surfactin against these strains. Compared with surfactin, iturin is thought to have more antifungal properties [31], and cyclodipeptides are known to be effective against *C. neoformans* [32].

Still other organisms were inhibited by 1B-23 itself, but not by its crude extract nor isolated surfactin, indicating that inhibition in these cases is caused by general mechanisms such as competition for resources, or by antimicrobial compounds that do not segregate with the crude extract. However, even in the presence of a pathogen insensitive to surfactin, surfactin still can play several important roles in in vivo biocontrol*.* For instance, its role in *Bacillus* biofilm formation, induction of systemic resistance in plants, and disruption of pathogen adhesion are critical to effective biocontrol, making surfactin an important predictor of their effectiveness [13, 15, 33]. The organisms that were inhibited by 1B-23 but not its crude extract or surfactin are *R. solani* (a basidiomycete mold), *Candida albicans* (an ascomycete yeast), *C. destructans*, *F. solani*, and *F. oxysporum* (ascomycete molds of the same family). Evidently, we cannot make any generalizations of fungal susceptibility to surfactin based on phylogeny. Our results suggest that surfactin is a major contributor to antimicrobial activity against several phytopathogens, and that surfactin-producing 1B-23 may be useful as a microbial pesticide.

Previous studies show that the production of lipopeptides can be affected by environmental factors including medium composition and moisture levels [34]. It is important to understand the effects of various growth conditions on production since the optimal growth conditions or growth curves may not necessarily be indicative of maximum production of antimicrobial compounds. In the current study, we looked at the effects of various temperatures and pH on surfactin production in 1B-23, as such knowledge could help determine the suitability of its use as a pesticide in various soil conditions. Initial results from cultures grown at various temperatures and harvested after 72 h found that the highest production occurred at 16 °C. This finding is consistent with studies on the related *B. amyloquefaciens* which also produces the most surfactin at around 15 °C [25, 26], while the more distantly related *B. subtilis* and *B. mojavensis* show maximum surfactin production at higher temperatures (≥30 °C) (e.g. [24, 25, 35].

For the data at various temperatures across multiple time points, we did not find a significant effect of temperature on surfactin production by 1B-23. It is possible that a significant effect would be detected with greater power (i.e. more replicates): the time course data had only four replicates per temperature while the initial data at 72 h, which showed significantly greater surfactin production at 16 °C versus 28 °C and 36 °C, had six replicates. Still, while the accumulated surfactin at 16 °C was not significantly greater, growth of 1B-23 at 16 °C was significantly lower versus the other temperature studied in the time course. Therefore 1B-23 grew less at 16 °C while accumulating equal or greater amounts of surfactin, meaning that individual 1B-23 cells must produce more surfactin, providing further evidence of surfactin upregulation. In addition, the mass of the crude extract, which contains surfactin and likely other hydrophobic antimicrobials, also increased at 16 °C. This suggests that surfactin is being upregulated without sacrificing the production of other important metabolites; a very important aspect for biocontrol. As a secondary metabolite, the lipoheptapepetide is produced at higher concentrations in circumstances of competition or abiotic stress [19]. It is then logical that surfactin would be upregulated under stress conditions in microbes, such as low growth temperatures [18]. However, the specific mechanisms underlying surfactin upregulation and efflux are still not well understood [36]. An implication of this study is that optimal growth of bacteria should not be used as an indication of maximum lipopeptide production. In the case of 1B-23, our data suggest decent surfactin production across several temperatures, even when bacterial growth is lower.

While maintaining the growth temperature at 16 °C and varying the pH of the media from 5 to 8, we found that the highest surfactin production occurred at pH 6, followed by pH 6.5. However, the only statistically significant difference was found between pH 6 and pH 8. While Meena et al. [22] found greater surfactin production by *B. subtilis* strain KLP2015 at higher pH (7–10), other studies found neutral or slightly acidic pH to favor the production of surfactin and fengycin [37, 38]. Additionally, pH 6 is the unofficial, but optimal growth pH for *B. velezensis* CBMB205^T^ as reported by Madhaiyan et al. [27].

Our results suggest that *B. velezensis* 1B-23 may be particularly useful in slightly acidic soils. Such conditions not only favour surfactin production, but the phytopathogens that were inhibited by purified surfactin from 1B-23 affect crops that also grow best in slightly acidic soils: *C. michiganensis* subsp. *michiganensis* affects tomato, *M. fructicola* affects stone fruits including apricot, cherry, peach, and plum, and *C. carbonum* affects apple, maize, and sorghum [39].

Studies of the related *B. amyloliquefaciens* show that surfactin production correlates with biocontrol effectiveness [19], and our previous research into the effects of 1B-23 on *C. michiganensis*-infected, greenhouse-grown tomatoes indicates that it can reduce disease symptoms in vivo, in a commercially relevant setting [28]. Still, while our focus for this study was on surfactin production by *Bacillus velezensis* 1B-23, it remains important to study the other antifungal metabolites and to test the effects on additional organisms in plant systems.

## Conclusions

Here, we showed that *B. velezensis* 1B-23 exhibits a high degree of inhibition against phylogenetically diverse fungal pathogens, and that some of this inhibition can be attributed to surfactin. We characterized *B. velezensis* 1B-23’s temperature- and pH-specific surfactin production, which to our knowledge has not previously been done in this species. Our results demonstrate that *B. velezensis* produces the most surfactin at lower temperatures (unlike many other *Bacillus* species) and at slightly acidic pH.

While the breadth of 1B-23’s antimicrobial activity makes it a possible contender as a commercial biocontrol agent, the optimal conditions for lipopeptide production and hence biocontrol can help to inform the specific situations where 1B-23 could be most useful. Due to the inconsistent effectiveness of microbial pesticides across different environments, it is imperative that such products be tailored to include a consortium of strains that are suited to the environments where they will be used. Our study indicates that 1B-23 can potentially contribute to biocontrol in slightly acidic soils of permanently or seasonally cool climates, such as Southern Ontario, Canada, from which this strain was isolated.

Overall, our research presents *B. velezensis* 1B-23 as a potential biocontrol agent to support sustainable and resilient agriculture, and paves the way for further optimization of surfactin production in *B. velezensis*. Such optimization may facilitate use of this species as a part of a microbial pesticide or as bioreactor for sustainable surfactin production.

## Methods

### *Isolation of B. velezensis* 1B-23

Isolation of *B. velezensis* 1B-23 was previously described [28]: Soil samples originating from the Blizman potato fields in Norfolk County, Ontario, Canada were collected during the summer of 2012. Over the previous three years, the soil was amended with bio-organic fertilizer each spring in an effort towards natural remediation. In the fourth and final year (2012), 10.0 g of moist soil was collected, suspended in 95 mL of sterile water, and shaken at room temperature for 10 min. The suspension was then serially diluted up to 10^− 10^, and dilutions were plated on tryptic soy agar (TSA) for 48 h at 28 °C to attain single microbial colonies. Permission for this research was obtained from the Canadian Food Inspection Agency (CFIA). 1B-23 was one of 600 bacterial isolates collected from the samples [40]. It was selected for further investigation due to its promising signs of antimicrobial activity during preliminary screening assays. The complete genome sequence has been submitted to the NCBI database (GenBank accession number: CP033967.1) and its full characterization are to be submitted elsewhere.

### In vitro assessment of antimicrobial properties

Bacterial pathogen (background) plates were prepared by first suspending the bacterial pathogen in 0.85% NaCl at an OD_600_ of 1.0 (~ 5 × 10^8^ CFU/mL). Three serial dilutions were prepared from this stock solution, ending with a final dilution factor of 10^3^. Each dilution was plated onto plates containing LB medium (10 g/L tryptone, 5 g/L yeast extract and 10 g/L NaCl; BioShop Canada Inc., Burlington, ON, Canada) with 15 g/L agar and allowed to dry prior to screening. Then, 0.5 mm discs of P8 Filter Paper (Thermo Fisher Scientific, Waltham, MA, USA) infused with 50 μL of 1B-23 (similarly prepared in 0.85% NaCl solution at an OD of 1.0) were placed onto the plates. For fungal pathogens, the background was prepared after 1B-23 was streaked in an open square pattern on LB agar plates, by placing a fungal plug in the center of the opened 1B-23 square. To assess the antimicrobial properties of the crude extract and surfactin, the pathogen backgrounds were prepared similarly with the following exceptions: (1) 50 μL of crude extract or surfactin (1.0, 2.5, 5.0, or 10 mg/mL) was used in place of 1B-23 to inoculate the filter discs and (2) a square whose corners were comprised by the crude extract (see methods below) or surfactin-inoculated filter discs were used to enclose the fungal plugs for antifungal assessment. Surfactin was isolated and purified from the crude extract using HPLC [40]; its identity was confirmed by de novo peptide sequencing with mass spectrometry (MS). Both crude extract and purified surfactin were suspended in methanol. All plates were parafilm-sealed and incubated for 48 h at 28 °C prior to imaging.

For negative controls, filter discs inoculated with the pathogens in 0.85% NaCl were used. To create the antimicrobial discs, the negative control discs were inoculated with methanol (Sigma-Aldrich, Mississauga, ON, Canada) to ensure that methanol alone was non-toxic to the pathogens.

### Surfactin production assays

For each experiment, 1B-23 was first grown in liquid LB medium, pH 6.8, and diluted to an OD_600_ of 0.01. The diluted culture was then separated into 100 mL aliquots, each placed into a 250 mL Erlenmeyer flask. Abiotic negative controls containing sterile LB broth were prepared alongside the samples. Flasks were placed in temperature-controlled incubators with shaking at 125 rpm for the specified time. The initial temperature study was done in replicates of six with one negative control for each temperature, and incubated for 72 h, at which time the OD_600_ was measured and the culture refrigerated (4 °C) until extraction of the hydrophobic metabolites. The time course experiments used four replicates and one negative control per temperature or pH. The pH was adjusted using HCl or NaOH. An aliquot of 10 mL was removed from each time course sample at each time point for extraction.

### Crude extract collection

Hydrophobic metabolites (crude extract) were collected from 10 mL aliquots of 1B-23 liquid cultures using a liquid-liquid extraction technique. The 1B-23 cultures were washed twice with ≥99.7% ethyl acetate (Sigma-Aldrich, Mississauga, ON, Canada), followed by collection of the organic phase and subsequent drying with 99% anhydrous sodium sulfate (Sigma-Aldrich, Mississauga, ON, Canada). The ethyl acetate was partly dried off using an IKA® RV10™ rotary evaporator (180 mBar and 125 rpm), followed by nitrogen gas to completion. The crude extract was then suspended in 100% methanol (Sigma-Aldrich, Mississauga, ON, Canada) to a final concentration of 10 mg/mL.

### Semi-quantification of the Total Surfactin content

Concentration-standardized (1 mg/mL) 1B-23 crude extract samples were subjected to high resolution mass analysis using an Agilent 1290 Ultra-High-Performance Liquid Chromatography (UHPLC; Agilent Technologies, Santa Clara, CA, USA) coupled to a Q-Exactive Quadrupole-Obitrap Mass Spectrometer (Thermo Fisher Scientific, Waltham, MA, USA). Analytes were separated on a C18 reverse phase column using a water-acetonitrile gradient with 0.1% formic acid, at a flow rate of 0.2 mL/min for over 7 min. We putatively identified the surfactins by analyte specific MS/MS diagnostic fragmentation pattern using XCalibur (Thermo Fisher Scientific). A linear standard curve based on purified surfactin C was made and used as a surrogate standard to semi-quantify all of the identified surfactin isoforms. For each replicate, the detected chromatographic areas of all surfactins were measured against the standard curve area to calculate the amount of all surfactins produced in a 1 mg/mL solution of crude extract. The calculated mass of surfactin per mg of crude extract was then multiplied by the total crude extract mass obtained from the original culture sample to compensate for the different amounts of extract obtained from each sample, and divided by the 10 mL volume of the extracted cell culture, to determine the mass of surfactin per mL of culture. From this value, we subtracted the mass of surfactin per mL obtained from the abiotic negative control, resulting in the semi-quantification of total surfactins per mL of 1B-23 cell culture.

### Statistical analysis

Initial differences in optical density and total surfactin mass (between treatments) were compared using one-way Analysis of variance (ANOVA) with the Bonferonni multiple comparison post-hoc test to limit Type 1 error.

For time course data, SPSS Statistics version 26 (IBM Corp., Armonk, New York, U.S.A.) was used to perform 2-way repeat-measures ANOVA, with the Sidak adjustment to limit Type 1 error for multiple comparisons (post-hoc test). A simple effects test to compare temperature or pH at each time point was prompted in the command syntax. Statistical significance was accepted where α < 0.05.

## Supplementary Information

**Additional file 1.**

**Additional file 2.**

## Data Availability

The datasets used and/or analysed during the current study are available from the corresponding author on reasonable request.

## Abbreviations

1B-23: *Bacillus velezensis* strain 1B-23
LB: Luria-Bertani medium for growth of bacteria
LC-MS: Liquid chromatography coupled to high resolution mass spectrometry
MS: Mass spectrometry
UHPLC: Ultra-high-performance liquid chromatography
