# Supplementary material for: Evaluating the biocontrol potential of Canadian strain Bacillus velezensis 1B-23 via its surfactin production at various pHs and temperatures
Source: BMC Biotechnol. 2021 Apr 29;21:31. doi: 10.1186/s12896-021-00690-x (PMC8082884; doi:10.1186/s12896-021-00690-x)

Evaluating the biocontrol potential of Canadian strain *Bacillus velezensis* 1B-23 via its surfactin production at various pHs and temperatures

Michelle S. M. Li, David A. Piccoli, Tim McDowell, Jacqueline MacDonald, Justin Renaud and Ze-Chun Yuan

Additional file 1.

In vitro antimicrobial activity of 1B-23 cell culture, crude extract, and purified surfactin against various microbial pathogens. Cell culture was suspended in 0.85 % NaCl at an OD_{600} of 1.0; while crude extract and surfactin were each suspended in 100 % methanol at 1.0, 2.5, 5.0 or 10 mg/mL.

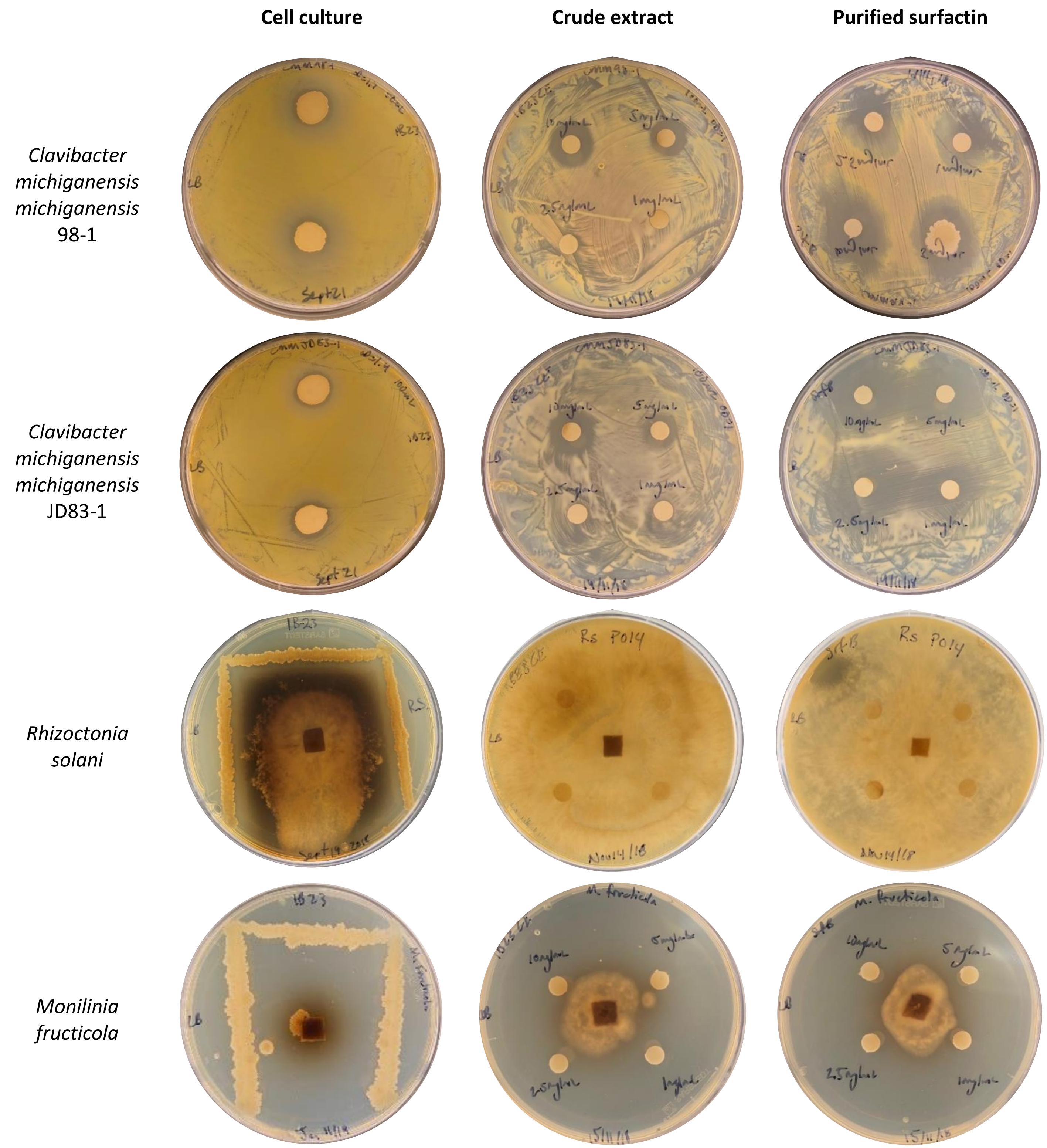
Cell culture
Crude extract
Purified surfactin
Clavibacter michiganensis michiganensis 98-1
Clavibacter michiganensis michiganensis JD83-1
Rhizoctonia solani
Monilinia fructicola

Cylindrocarpon destructans 1666

Cochliobolus carbonum

Fusarium solani

Fusarium oxysporum

Cryptococcus neoformans var. *grubii* H99

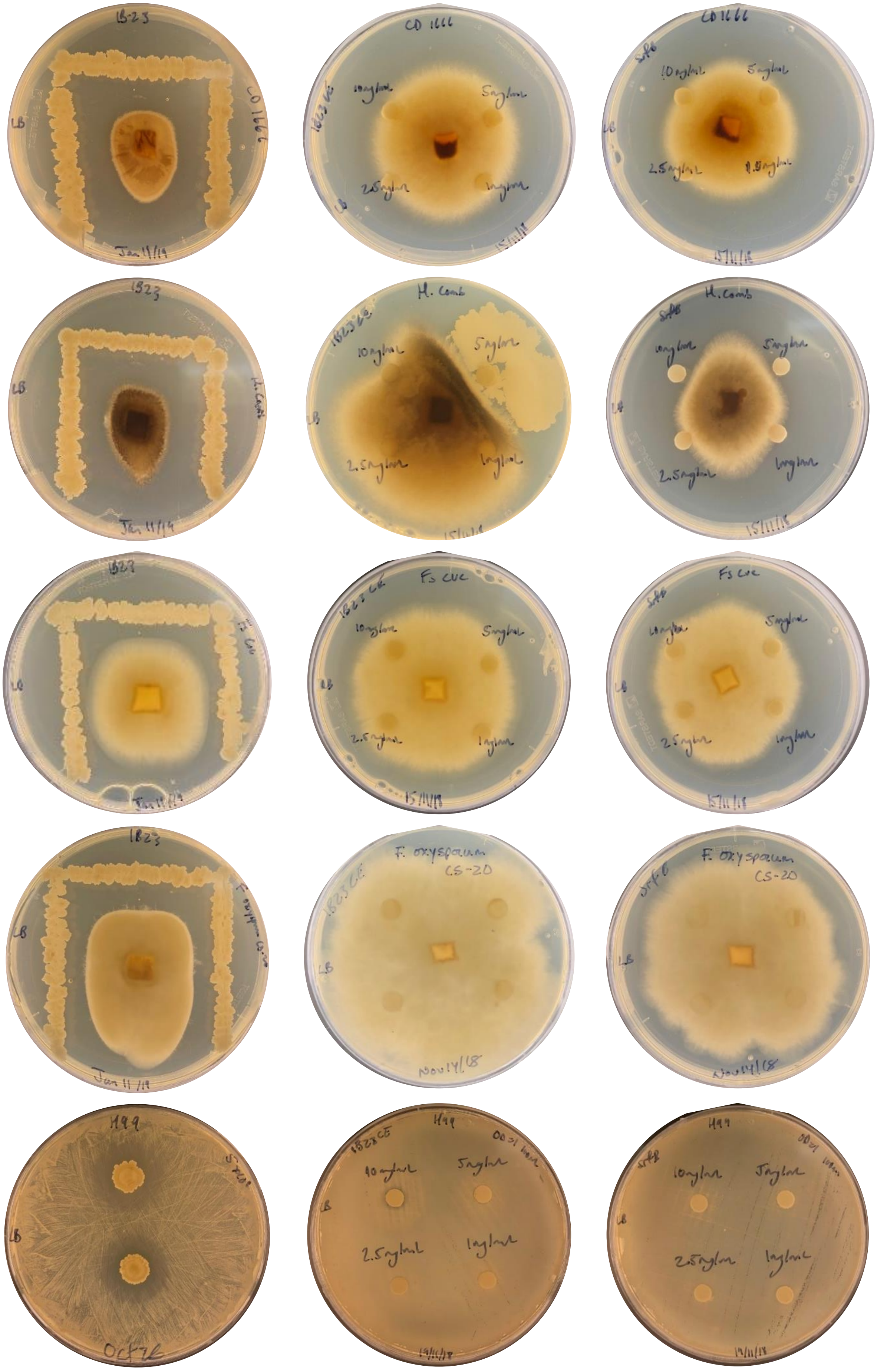

Cryptococcus neoformans var. *neoformans* JEC20

Cryptococcus neoformans var. *neoformans* Y290.90

Candida albicans ARG100

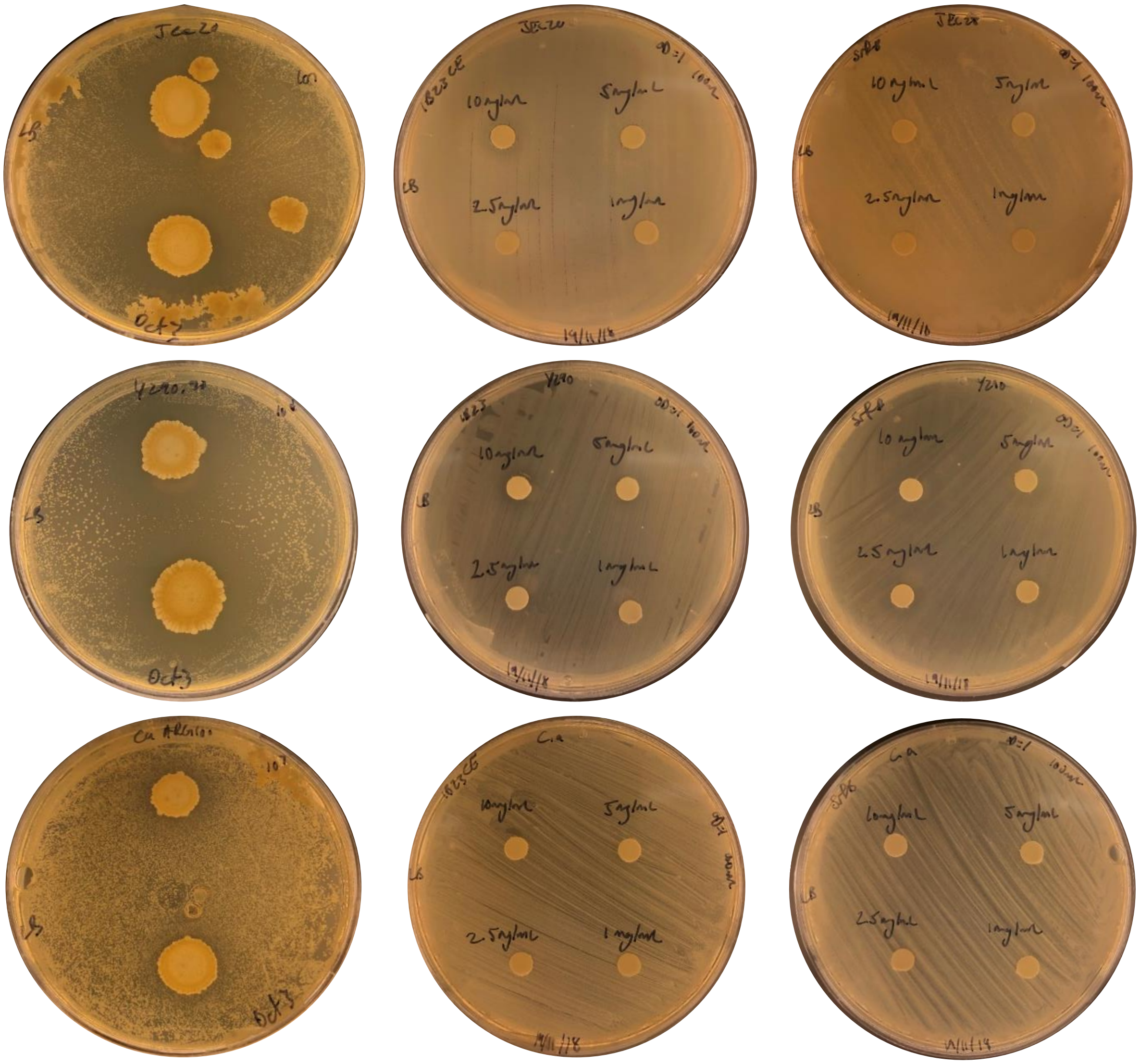

Supplement: Supplementary file 1 — Additional file 1. [file 12896_2021_690_MOESM1_ESM.pdf]
