# Supplementary material for: Evaluating the biocontrol potential of Canadian strain Bacillus velezensis 1B-23 via its surfactin production at various pHs and temperatures
Source: BMC Biotechnol. 2021 Apr 29;21:31. doi: 10.1186/s12896-021-00690-x (PMC8082884; doi:10.1186/s12896-021-00690-x)
Supplement: Supplementary file 2 — Additional file 2. [file 12896_2021_690_MOESM2_ESM.pdf]

Evaluating the biocontrol potential of Canadian strain *Bacillus velezensis* 1B-23 via its surfactin production at various pHs and temperatures

Michelle S. M. Li, David A. Piccoli, Tim McDowell, Jacqueline MacDonald, Justin Renaud and Ze-Chun Yuan

Additional file 2.

Antimicrobial components of the *B. velezensis* 1B-23 crude extracts. 1B-23 cultures grown at (a) 28 °C, (b) 24 °C, (c) 20°C, and (d) 16°C were screened by high resolution MS. Surfactins were the major lipopeptide class detected. Epoxy-macrolactin A and cyclic dipeptides were also detected in the extracts. Minor amounts of iturin lipopeptides were also detected. Note that relative abundance can only be compared across temperatures and not across components due to differences in ionization efficiency for different molecules. Structures of the detected surfactins (surfactin B, surfactin C, and surfactin D) are shown below the plot. The full chemical structures (left) were created using Marvin sketch, version 20.10.0 (ChemAxon Ltd., Budapest, Hungary).

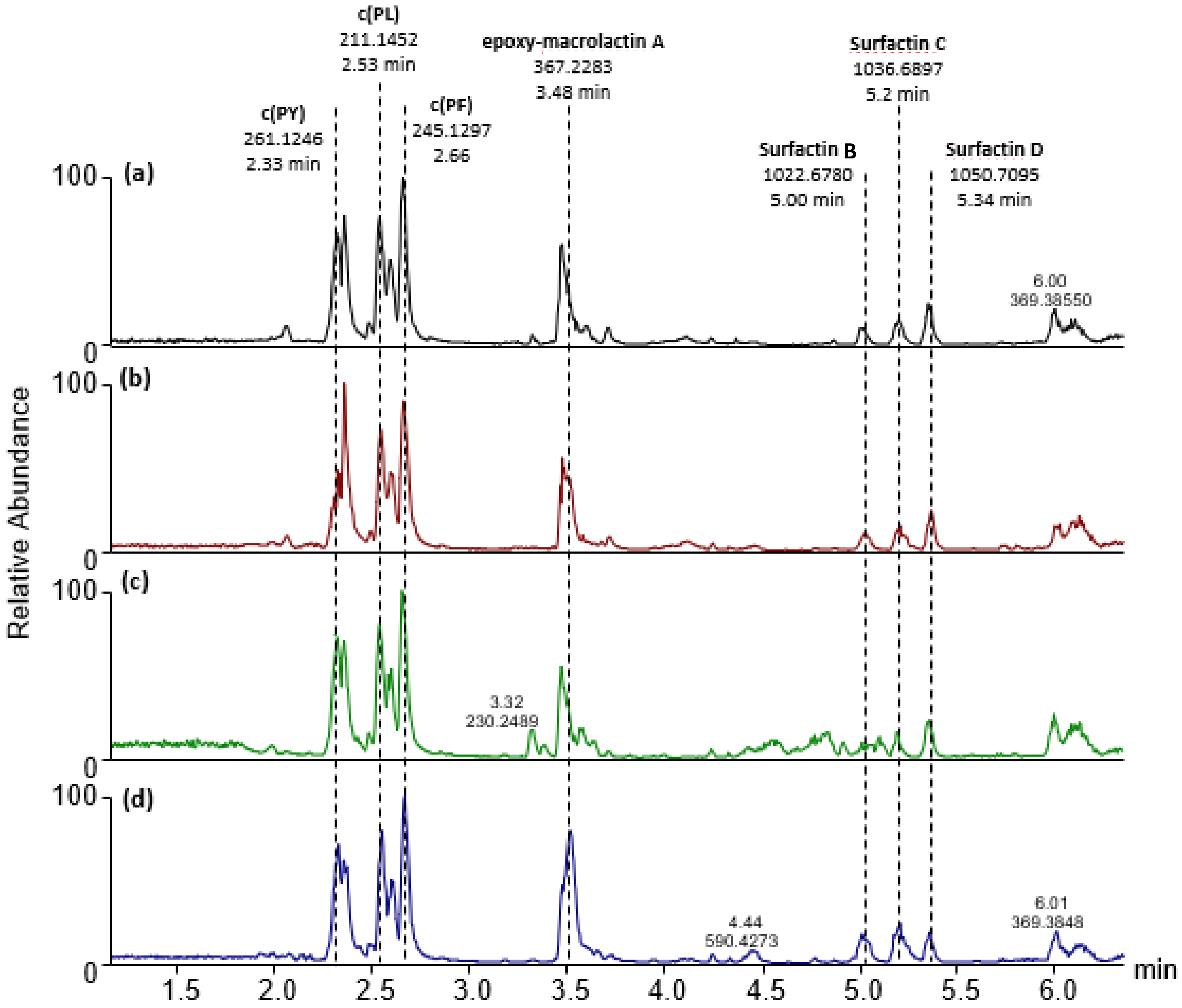

c(PY)
261.1246
2.33 min
c(PL)
211.1452
2.53 min
c(PF)
245.1297
2.66
epoxy-macrolactin A
367.2283
3.48 min
Surfactin B
1022.6780
5.00 min
Surfactin C
1036.6897
5.2 min
Surfactin D
1050.7095
5.34 min
(a)
(b)
(c)
(d)
6.00
369.38550
3.32
230.2489
4.44
590.4273
6.01
369.3848
Relative Abundance
100
0
1.5
2.0
2.5
3.0
3.5
4.0
4.5
5.0
5.5
6.0
min

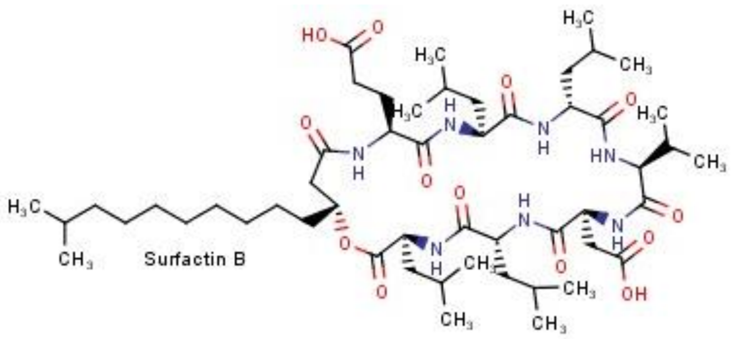
Surfactin B

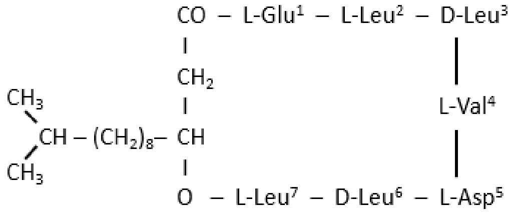
CO – L-Glu1 – L-Leu2 – D-Leu3
CH2
CH3
CH – (CH2)8– CH
CH3
L-Val4
O – L-Leu7 – D-Leu6 – L-Asp5

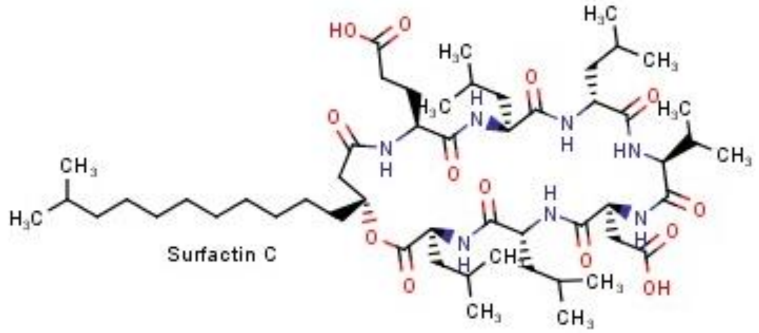
Surfactin C

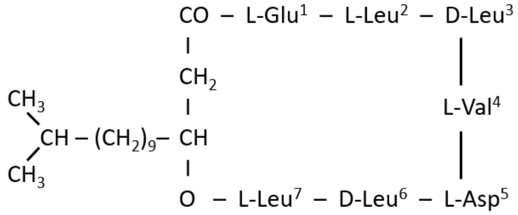
CO – L-Glu1 – L-Leu2 – D-Leu3
CH2
CH3
CH – (CH2)9– CH
CH3
L-Val4
O – L-Leu7 – D-Leu6 – L-Asp5

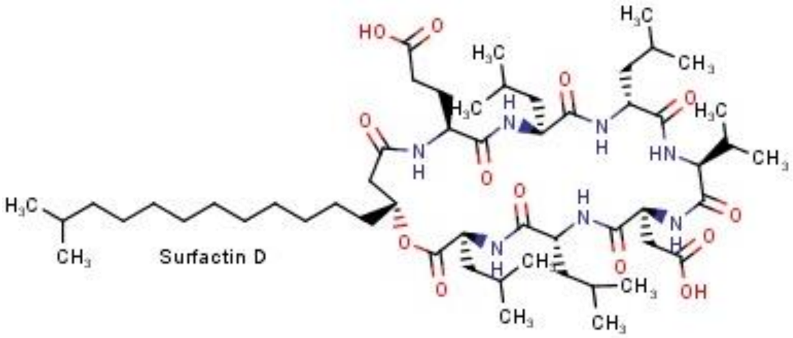
Surfactin D

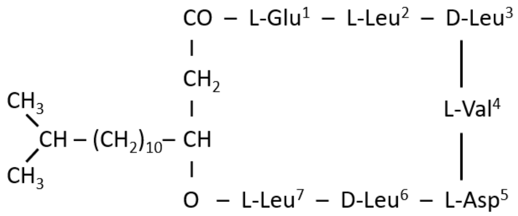
CO – L-Glu1 – L-Leu2 – D-Leu3
CH2
CH3
CH – (CH2)10– CH
CH3
L-Val4
O – L-Leu7 – D-Leu6 – L-Asp5
